# Supplementary material for: Evaluation of DNA Methylation Array for Glioma Tumor Profiling and Description of a Novel Epi-Signature to Distinguish IDH1/IDH2 Mutant and Wild-Type Tumors
Source: Genes (Basel). 2022 Nov 9;13(11):2075. doi: 10.3390/genes13112075 (PMC9690723; doi:10.3390/genes13112075)
Supplement: Supplementary file 1 [file genes-13-02075-s001.zip › Schenkel et al_2022_Supplemental Table S1_Final.pdf]

Supplementary Table S1:Demographic and histophatological information of glioma tissues samples

**Sample #**	**Age**	**Gender**	**Original Histopathological classification**
**1**	40	M	Oligodendroglioma, grade II
**2**	37	F	Oligodendroglioma, grade II
**3**	54	F	Oligodendroglioma, grade II
**4**	43	F	Oligodendroglioma, grade II
**5**	67	F	Oligodendroglioma, grade III
**6**	58	M	Oligodendroglioma, grade III
**7**	82	F	Oligodendroglioma, grade III
**8**	48	F	Oligodendroglioma, grade III
**9**	54	M	Oligodendroglioma, grade III
**10**	60	F	Oligodendroglioma, grade III
**11**	34	M	Oligodendroglioma, grade III
**12**	58	M	Oligodendroglioma, grade III
**13**	75	M	Glioblastoma
**14**	59	M	Glioblastoma
**15**	73	F	Glioblastoma
**16**	59	M	Glioblastoma
**17**	37	M	Glioblastoma
**18**	53	M	Glioblastoma
**19**	65	M	Glioblastoma
**20**	50	M	Glioblastoma

**21**	40	M	Oligoastrocytoma, grade II
**22**	27	M	Oligoastrocytoma, grade II
**23**	48	F	Oligoastrocytoma, grade II
**24**	30	M	Oligoastrocytoma, grade II
**25**	42	M	Oligoastrocytoma, grade II
**26**	64	M	Oligoastrocytoma, grade III
**27**	46	M	Oligoastrocytoma, grade III
**28**	64	M	Oligoastrocytoma, grade III
**29**	68	M	Oligoastrocytoma, grade III
**30**	46	F	Oligodendroglioma, grade II
**31**	60	M	Oligodendroglioma, grade III
**32**	64	M	Oligodendroglioma, grade III
**33**	47	M	Glioblastoma
**34**	48	F	Oligoastrocytoma, grade II
**35**	67	M	Glioblastoma
**36**	39	M	Oligoastrocytoma, grade II
**37**	57	M	Oligoastrocytoma, grade III
**38**	79	M	Oligoastrocytoma, grade III
**39**	62	M	Oligoastrocytoma, grade III
**40**	68	F	Glioblastoma
**41**	71	F	NA
**42**	54	F	Oligodendroglioma, grade III

**43**	69	M	Glioblastoma
**44**	45	M	Oligodendroglioma, grade II
**45**	32	F	Oligodendroglioma, grade III
**46**	71	M	Glioblastoma
**47**	60	M	Oligodendroglioma, grade II
**48**	53	F	Oligodendroglioma, grade III

Clinical samples (replication cohort) are underlined. F=female; M=male. NA=Not available/Not applicable
